# Editorial: Quantifying and optimizing elite performance through cognitive neuroscience

**DOI:** 10.3389/fspor.2026.1882076

**Published:** 2026-06-05

**Authors:** Tao Song

**Affiliations:** School of Psychology, Shanghai University of Sport, Shanghai, China

**Keywords:** cognitive neuroscience, elite performance, mental fatigue, neuroplasticity, sustained attention

The pursuit of excellence in elite sports has long focused on physical conditioning, technical skill, and tactical acumen. However, a growing body of evidence highlights that the brain—its attentional resources, neural efficiency, and cognitive resilience—is the ultimate arbiter of performance under pressure. This Research Topic sought to explore and integrate emerging evidence toward a working framework for quantifying sport-specific neurocognitive functions and translating these insights into actionable training strategies. The contributions assembled here span meta-analytic brain mapping, ecologically valid assessment of attention, the overlooked phenomenon of mental fatigue in Olympic combat sports, an empirical examination of mental fatigue in Paralympic boccia, and a novel movement-sequencing intervention grounded in motor development principles. Collectively, they advance the field from descriptive observations toward predictive models and evidence-based interventions.

## Mapping the athlete's brain network

Wang et al. provide a critical foundation through an activation likelihood estimation (ALE) meta-analysis and meta-analytic connectivity modeling (MACM) of motor imagery and action anticipation. Their findings reveal that athletes recruit a more efficient fronto-parietal-temporal network compared to non-athletes, who rely more heavily on visual regions. This integrated network supports both internally generated (imagery) and externally guided (anticipation) action processes, offering neural markers of perceptual-motor expertise. By quantifying the brain's structural and functional reorganization with long-term training, this work establishes candidate neural biomarkers for assessing elite performance—a key step toward objective neurocognitive profiling.

## Refining assessment: sustained attention in context

Blumberg et al. tackle the challenge of quantifying attention, a core cognitive faculty in elite sport. Moving beyond traditional between-group comparisons, they employ a novel within-subjects variance time-course procedure to examine sustained attention in 198 elite athletes across team, speed-strength, and precision-skill sports. Their results show that team sport athletes exhibit superior accuracy on the Sustained Attention to Response Task (SART), but more importantly, they reveal that athletes fluctuate between “in the zone” and “out of the zone” attentional states. This idiographic approach captures the dynamic nature of attention under sport-specific demands, providing a more precise assessment tool that can guide individualized cognitive training regimens.

## Unveiling mental fatigue as a performance determinant

Bian et al. shift the focus to an often-neglected dimension of elite performance—mental fatigue. In their opinion article on Olympic combat sports, they synthesize evidence from judo, taekwondo, and fencing, showing that while physical fatigue has been extensively studied, the cognitive toll of high-intensity, intermittent competition remains underexplored. Notably, fencing research has begun to quantify mental fatigue accumulation across consecutive matches and to test brain endurance training (BET) as a countermeasure. The authors call for ecologically valid protocols that replicate real-world work-to-rest ratios, decision-making pressures, and environmental distractions. Their work underscores that optimizing elite performance requires not only enhancing cognitive capacities but also managing cognitive load and fatigue resilience.

da Silva et al. provide an empirical investigation of mental fatigue in Paralympic boccia. Eleven athletes (six with cerebral palsy, five without) underwent three experimental conditions: mental fatigue induction, low cognitive effort, and control. Accuracy was assessed at distances of 3, 6, and 9 m. Contrary to the broader expectation that mental fatigue impairs performance, the results showed no significant effect of either mental fatigue or low cognitive effort on accuracy. This null finding is striking and important: it suggests that the impact of mental fatigue may be sport-specific, population-specific, or task-specific. While Bian et al. highlight the need to manage mental fatigue in dynamic, time-constrained combat sports, da Silva et al. remind us that not all precision-based, self-paced sports are equally susceptible. Together, these two contributions underscore that the relationship between mental fatigue and elite performance is more nuanced than previously assumed.

## Translating theory into practice: movement sequences from motor development

Msaidie et al. offer a practical intervention grounded in neurocognitive principles. Their narrative review proposes sequences of combinations of movements (SCM) based on early motor development stages (MDS), arguing that these foundational patterns—posture, balance, coordination—can refine sensorimotor integration and neuroplasticity in elite athletes. While currently untested in high-performance sport, the SCM framework provides testable hypotheses for warm-up, injury prevention, and recovery protocols. This work bridges the gap between developmental neuroscience and applied training, exemplifying the translational goal of our Research Topic.

Together, these five contributions illuminate a pathway from quantification to optimization, while also revealing important boundary conditions. First, quantifying elite performance demands multi-level approaches: from large-scale brain networks (Wang et al.) to within-person attentional dynamics (Blumberg et al.) and real-time fatigue monitoring (Bian et al.). Second, the effects of mental fatigue are not uniform: Bian et al. demonstrate its accumulation and negative impacts in Olympic combat sports, whereas da Silva et al. show no detrimental effect on accuracy in Paralympic boccia. This contrast invites future research to identify moderating factors—such as sport type (interactive vs. self-paced), disability status, task complexity, and cognitive load intensity. Third, optimization emerges from interventions that respect the brain's plastic potential—whether through cognitive training (BET), movement re-education (SCM), or tailored attentional drills. A recurring insight is the necessity of ecological validity: laboratory tasks must mirror the complexity, time pressure, and contextual distractions of actual competition.

Future research should integrate these strands. For instance, combining neuroimaging with longitudinal tracking of mental fatigue could reveal how neural efficiency degrades under cumulative cognitive load. Comparative studies across different sport populations—including athletes with disabilities—could clarify why mental fatigue affects some groups more than others. Similarly, SCM protocols could be evaluated using the within-subjects attentional metrics developed by Blumberg et al. Interdisciplinary collaborations—neuroscience, biomechanics, psychophysiology, and coaching—will be essential to move from proof-of-concept to scalable solutions. The journey from quantifying the elite brain to optimizing its performance has only just begun.

